# The Great Capacity on Promoting Melanogenesis of Three Compatible Components in *Vernonia anthelmintica (L.) Willd.*

**DOI:** 10.3390/ijms22084073

**Published:** 2021-04-15

**Authors:** Yifan Lai, Qingyuan Feng, Rui Zhang, Jing Shang, Hui Zhong

**Affiliations:** 1 State Key Laboratory of Natural Medicines, China Pharmaceutical University, Nanjing 210009, China; Yifan_Lai@outlook.com (Y.L.); 15651715868@163.com (Q.F.); zhangrui19950825@163.com (R.Z.); 2 Jiangsu Key Laboratory of TCM Evaluation and Translational Research, China Pharmaceutical University, Nanjing 211198, China

**Keywords:** melanogenesis, ROS, inflammatory, zebrafish, butin, caffeic acid, luteolin, synergistic effect

## Abstract

To investigate a possible methodology of exploiting herbal medicine and design polytherapy for the treatment of skin depigmentation disorder, we have made use of *Vernonia anthelmintica (L.) Willd.*, a traditional Chinese herbal medicine that has been proven to be effective in treating vitiligo. Here, we report that the extract of *Vernonia anthelmintica (L.) Willd*. effectively enhances melanogenesis responses in B16F10. In its compound library, we found three ingredients (butin, caffeic acid and luteolin) also have the activity of promoting melanogenesis in vivo and in vitro. They can reduce the accumulation of ROS induced by hydrogen peroxide and inflammatory response induced by sublethal concentrations of copper sulfate in wild type and green fluorescent protein (GFP)-labeled leukocytes zebrafish larvae. The overall objective of the present study aims to identify which compatibility proportions of the medicines may be more effective in promoting pigmentation. We utilized the D-optimal response surface methodology to optimize the ratio among three molecules. Combining three indicators of promoting melanogenesis, anti-inflammatory and antioxidant capacities, we get the best effect of butin, caffeic acid and luteolin at the ratio (butin:caffeic acid:luteolin = 7.38:28.30:64.32) on zebrafish. Moreover, the effect of melanin content recovery in the best combination is stronger than that of the monomer, which suggests that the three compounds have a synergistic effect on inducing melanogenesis. After simply verifying the result, we performed in situ hybridization on whole-mount zebrafish embryos to further explore the effects of multi-drugs combination on the proliferation and differentiation of melanocytes and the expression of genes (*tyr*, *mitfa*, *dct*, *kit*) related to melanin synthesis. In conclusion, the above three compatible compounds can significantly enhance melanogenesis and improve depigmentation in vivo.

## 1. Introduction

Skin pigmentation is an important human phenotypic trait whose regulation is related to many factors. The pigment melanin is produced by melanocytes in a complex process called melanogenesis. It is a physiological process leading to the production of melanin pigment and a crucial step for the regulation of melanocyte functions, including photoprotection [1,2,3]. The melanocyte interacts with endocrine, immune, inflammatory and central nervous systems, and its activity is also regulated by extrinsic factors such as ultraviolet radiation and drugs [4].

The skin is susceptible to oxidative damage, and reactive oxygen species (ROS) are an important by-product of melanin synthesis [5]. However, ROS production has been shown to suppress pigmentation in pigment cells and accumulated in the epidermis, which modulates the expression of the key pigment “melanin” synthesizing enzymes and cell damage [6,7]. Further, increased levels of ROS in melanocytes may cause defective apoptosis resulting in release of aberrated proteins, which can lead to inflammation [8]. The intracellular levels of H2O2 and other ROS also increase in response to cytokines such as TNF-α and TGF-β1, which are potent inhibitors of melanogenesis [9]. Moreover, inflammation-associated pigmentation changes are extremely common, for example, IL-17 and TNF synergistically modulate cytokine expression while suppressing melanogenesis [10]. However, in any case, maintaining the balance of pigment regulation is the ultimate goal.

Vitiligo is an acquired depigmenting disease manifested by chalk-or milk-white colored macules of several millimeters to centimeters in diameter [11]. It affects nearly 100 million people around the world and has regional and ethnic differences. The pathogenic factors mainly include genetics, mental stress, autoimmunity, neurochemical factors, oxidative stress, endocrine, melanocyte shedding, but not yet clear [12,13]. Up to now, the only drug approved by the FDA for the treatment of vitiligo is monobenzone cream, and this drug is the reverse treatment of vitiligo. Drugs are used for depigmentation treatment of patients with vitiligo whose skin area is more than 50% and hyperpigmentation diseases such as melanoma [14]. Therefore, there is no specific treatment for vitiligo at present. Due to the loss of functional melanocytes, restoring cell numbers and promoting melanin synthesis is a key strategy for the treatment of vitiligo.

*Vernonia anthelmintica (L.) Willd*. has a long history of traditional use for the management of several disorders related to skin, central nervous system, kidney, gynecology, gastrointestinal, metabolism and general health, especially in the treatment of vitiligo for thousands of years in China [15,16]. Although the anti-vitiligo mechanisms remain ambiguous, the herb has been universally used in Uyghur hospitals to treat vitiligo. To date, about 20 biologically active compounds of *Vernonia anthelmintica (L.) Willd.* were collected from the TCMID database, the Chinese Academy of Sciences Chemistry Database and the TCMSP database including flavonoids, caffeic acid-based quinines, sesquiterpenoids and steroids, etc [17]. Evidence suggests that ethanol seed extract induces melanogenesis by increasing the expression of TYR, TRP-1, TRP-2 and MITF in B16 cells, and moreover, it exhibits significant in vitro antioxidant and in vivo anti-inflammatory potential [18,19]. Butin, as one of the most important active compounds in *Vernonia anthelmintica (L.) Willd.*, plays a therapeutic effect by increasing the expression of TYR and TRP-1 protein and reducing the activity of MDA and CHE in a mouse model of hydroquinone-induced vitiligo [20]. Equivalently, more active compounds are isolated and identified such as isorhamnetin, kaempferide, 1,5-dicaffeoylquinic acid and benzoyl-vernovan, etc. They can significantly increase the expression of melanin-biosynthetic genes (MC1R, MITF, TYR, TYRP1 and DCT) and the tyrosinase activity with multiplex signaling pathways [21,22,23]. However, the current anti-vitiligo treatment for *Vernonia anthelmintica (L.) Willd.* is still at the stage of discovery of the material basis and the mechanism, and no monomer or component has been found to be more effective than the extract.

Here, we verified the melanogenesis, anti-inflammatory and anti-oxidation effects of butin and found two newly discovered compounds with similar effects, caffeic acid and luteolin in B16F10 cells and zebrafish. What is more, when they are combined with butin, they can synergeticly enhance the effect of melanogenesis. We performed in situ hybridization on whole-mount zebrafish embryos to further explore the effects of multi-drug combination on the proliferation and differentiation of melanocytes and the expression of genes (*tyr*, *mitfa*, *dct*, *kit*) related to melanin synthesis.

## 2. Results

### 2.1. Cytotoxicity toward B1610 Cells

**Figure 1:**
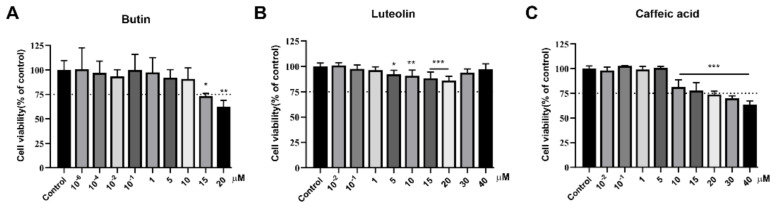
Cytotoxicity of compounds toward B1610 Cells. (**A**) B16 cells incubated with various concentrations [10^−6^, 10^−4^, 10^−2^, 10^−1^, 1, 5, 10, 15, 20] μmol·L^−1^ of butin for 48 h; (**B**,**C**) B16 cells incubated with various concentrations of luteolin and caffeic acid for 48 h. cell viability was determined using an MTT assay. Results shown are mean ± SEM and are representative of three independent experiments. Data were analyzed by one-way analysis of variance (ANOVA) followed by post hoc Tukey test. * *p* < 0.05, ** *p* < 0.01, *** *p* < 0.001 vs. Control. The dotted line indicates the relative viability of 75% of the cells.

To determine whether butin, caffeic acid and luteolin have cytotoxic effects, we treated B16F10 cells with these compounds at various concentrations; cell viability was determined using the MTT assay. As shown in Figure 1, the compounds were found to be non-toxic at concentrations ranging: butin 0–10 μmol·L^−1^, luteolin 0–1 μmol·L^−1^ and caffeic acid 0–5 μmol·L^−1^.

### 2.2. Melanin Content Assays in B16F10 Cells

**Figure 2:**
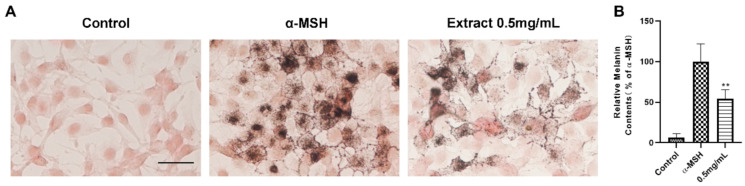
Extract of *Vernonia anthelmintica (L.) Willd.* induces melanogenesis. (**A**) Masson-Fontana melanin ammonia silver stain assay to test extract of *Vernonia anthelmintica (L.) Willd.* and α-MSH (20 nM) melanogenesis effect on B16F10 cells contrast to Control. Cells were treated for 48 h. The scale bars represent 50 μm. (**B**) Collecting B16 cells in each group for testing melanin content (relative melanin contents % of α-MSH), results shown are mean ± SEM. Data were analyzed by ANOVA followed by post hoc Tukey test, ** *p* < 0.01 vs. α-MSH.

As mentioned previously, ethanol seed extract of *Vernonia anthelmintica* induces melanogenesis by multiple ways. We determined the effect of extract (0.5 mg·mL^−1^) of *Vernonia anthelmintica (L.) Willd.* in B16F10 cells. It suggests that the seed can significantly promote melanogenesis in vitro (Figure 2).

**Figure 3:**
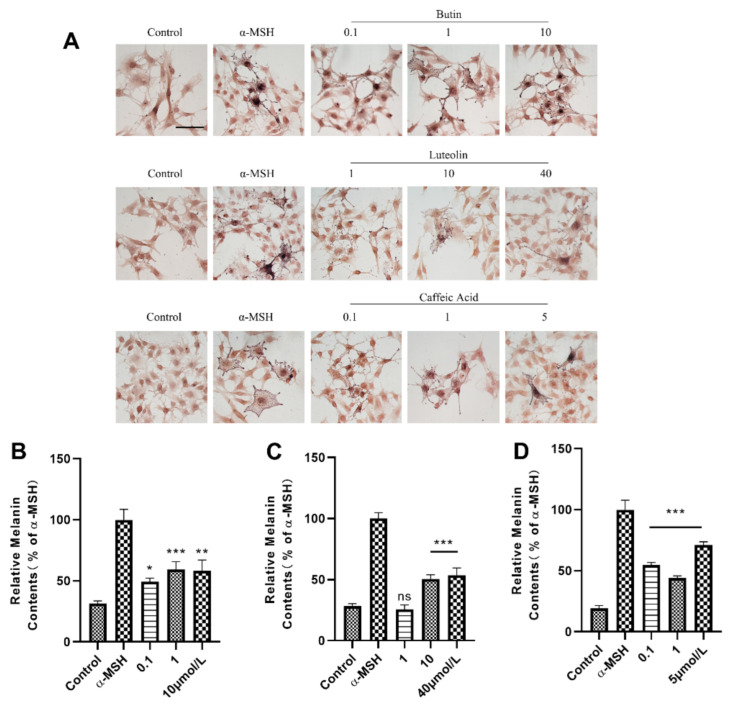
Compounds promote melanin synthesis on B16F10 cells. (**A**) Cells incubated with various concentration of butin [0.1, 1, 10] μmol·L^−1^, luteolin [1, 10, 40] μmol·L^−1^ and caffeic acid [0.1, 1, 5] μmol·L^−1^ to test melanogenesis effect contrast to α-MSH (20 nM) group and control group. Cells were treated for 48 h. The scale bars represent 30 μm. (**B**–**D**) are melanin contents (relative melanin contents % of α-MSH) in butin, luteolin and caffeic acid groups, results shown are mean ± SEM. Data were analyzed by ANOVA followed by post hoc Tukey test, ns, non-significant, * p < 0.05, ** *p* < 0.01, *** *p* < 0.001 vs. α-MSH.

To examine these compounds against B16 cell lysate to identify their influence on stimulation of melanin pigmentation. We also utilized the method of Masson-Fontana melanin ammonia silver stain to demonstrate the effect of drugs to promote melanin synthesis. As the results shown in Figure 3, the melanin contents were significantly increased as the concentration increases within a certain range. However, α-MSH, as a natural source agent, shows the best effects in an exceptionally low concentration. Furthermore, butin at 10 μmol·L^−1^ was identified as the most potent compound in vitro, and the effects of luteolin and caffeic acid are slightly inferior.

### 2.3. Effect of Compounds on Melanogenesis In Vivo

We utilized zebrafish to test the effect of compounds on melanogenesis, and prior to the investigation of melanogenesis, a toxicity assay was performed to determine the toxicity of the selected compounds to zebrafish. A significantly lower toxicity rate was observed in embryos at the following concentrations: butin 0–150 μmol·L^−1^, caffeic acid 0–200 μmol·L^−1^, luteolin 0–80 μmol·L^−1^ (the concentration is related to its maximum solubility), for 48 h indicating their optimal effective concentration toward zebrafish embryos.

**Figure 4:**
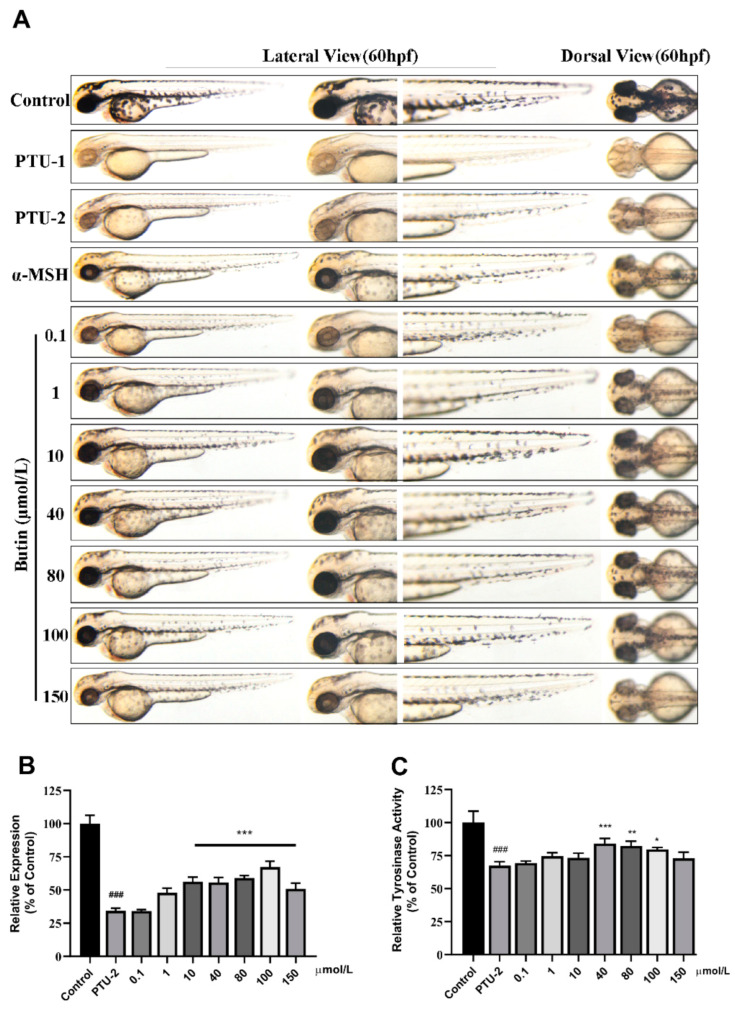
Effect of butin on melanogenesis in vivo. (**A**) Zebrafish melanin phenotype in various concentrations of butin [0.1, 1, 10, 40, 80, 100 and 150] μmol·L^−1^, the α-MSH is 60 μmol·L^−1^, and the PTU-1 group was not washed at all while the PTU-2 group was washed before administration. The control, PTU-1, PTU-2 and α-MSH groups share with caffeic acid and luteolin. (**B**) Collecting zebrafish in each group for testing melanin content (relative expression % of control). (**C**) Tyrosinase activity in zebrafish exposure to butin for 24 h. In (**B**,**C**), results shown are mean ± SEM. Data were analyzed by ANOVA followed by post hoc Tukey test. n = 30 zebrafish, ^###^ *p*<0.001 vs. Control, *** *p* < 0.001, ** *p* < 0.01, * *p* < 0.05 vs. PTU-2.

**Figure 5:**
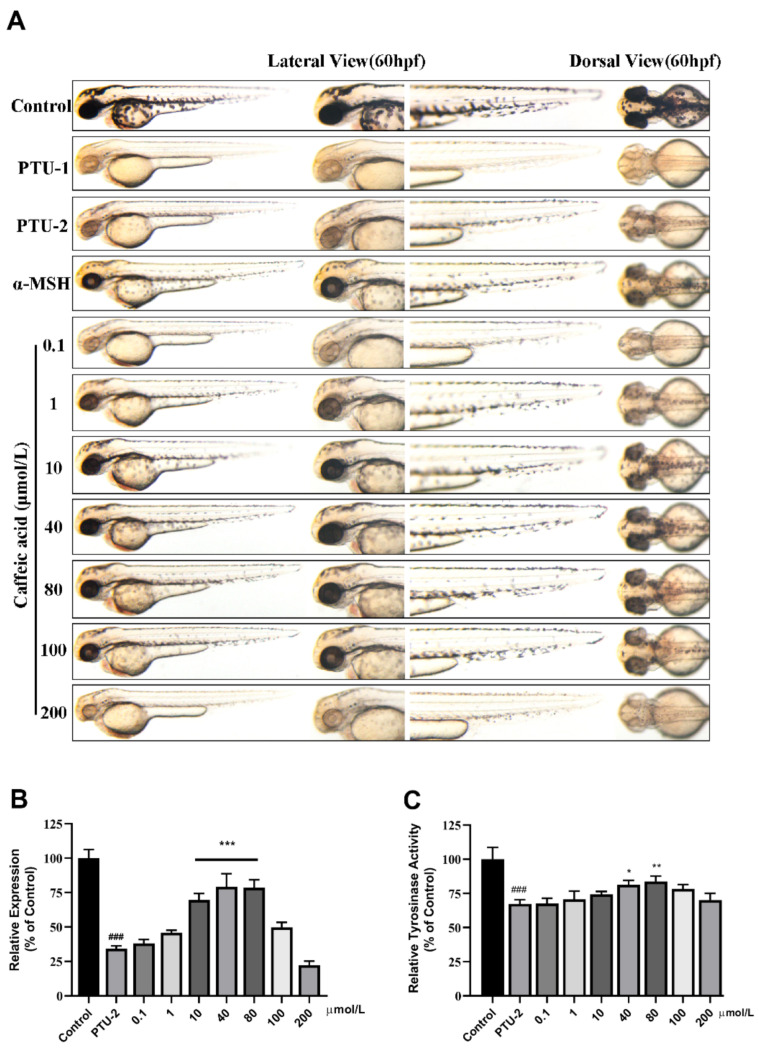
Effect of caffeic acid on melanogenesis in vivo. (**A**) Zebrafish melanin phenotype in various concentrations of caffeic acid [0.1, 1, 10, 40, 80, 100 and 200] μmol·L^−1^, the α-MSH is 60 μmol·L^−1^, and the PTU-1 group was not washed at all while the PTU-2 group was washed before administration. (**B**) Collecting zebrafish in each group for testing melanin content (relative expression % of control). (**C**) Tyrosinase activity in zebrafish exposure to butin for 24 h. In (**B**,**C**), results shown are mean ± SEM. Data were analyzed by ANOVA followed by post hoc Tukey test. n = 30 zebrafish, ^###^ *p* < 0.001 vs. Control, *** *p* < 0.001, ** *p* < 0.01, * *p* < 0.05 vs. PTU-2.

**Figure 6:**
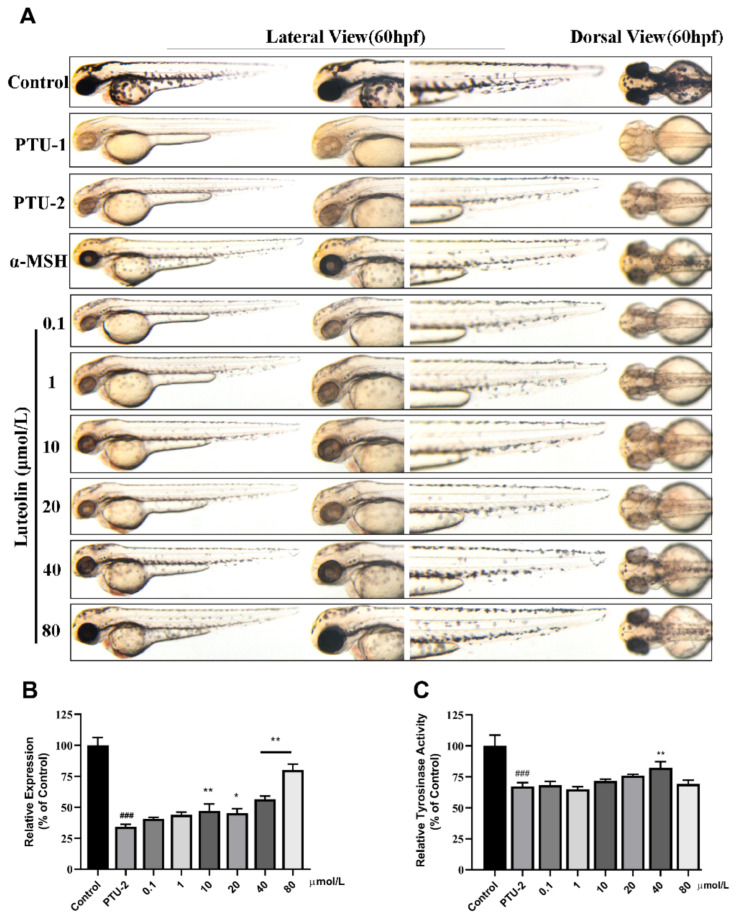
Effect of luteolin on melanogenesis in vivo. (**A**) Zebrafish melanin phenotype in various concentrations of luteolin [0.1, 1, 10, 40 and 80] μmol·L^−1^, the α-MSH is 60 μmol·L^−1^, and the PTU-1 group was not washed at all while the PTU-2 group was washed before administration. (**B**) Collecting zebrafish in each group for testing melanin content (relative expression % of control). (**C**) Tyrosinase activity in zebrafish exposure to butin for 24 h. In B and C, results shown are mean ± SEM. Data were analyzed by ANOVA followed by post hoc Tukey test. n = 30 zebrafish, ^###^ *p* < 0.001 vs. Control, ** *p* < 0.01, * *p* < 0.05 vs. PTU-2.

Pigmentation was analyzed by photography as shown in Figure 4, Figure 5 and Figure 6. As shown in Figure 4A lateral and dorsal view, the melanin content of zebrafish first increases and then decreases as the concentration of butin increases, and exhibited excellent results at a concentration of 10, 40, 80 and 100 μmol·L^−1^. When the concentration continues to increase, melanin synthesis is inhibited, possibly due to the toxicity of the compound. It suggests that the highest melanin content is up to ~65%. What is more, the result of tyrosinase activity is consistent with melanin, except that its highest point appears at 40 μmol·L^−1^ (Figure 4C).

Then we investigated the effect of caffeic acid on melanogenesis in vivo (Figure 5). As the same operation as butin, we found that the melanin content of zebrafish first increases and then decreases as the concentration of compound increases, and exhibited excellent results at a concentration of 10, 40 and 80 μmol·L^−1^. This performance is incredibly significant, means that caffeic acid has a narrower onset range. However, we did not see it had a big impact on tyrosinase activity except in 40 and 80 μmol·L^−1^.

Luteolin is a potential compound from *Vernonia anthelmintica (L.) Willd.* in inducing melanogenesis. However, the highest concentration we could get is 80 μmol·L^−1^ because its solubility in water has certain limitations. The results shown in Figure 6A, within the allowable range of solubility, the effect increases with increasing concentration, and it exhibits excellent results at a concentration of 80 μmol·L^−1^ (Figure 6B). While the 40 μmol·L^−1^ of concentration highly up-regulated compared with PTU-2 (Figure 6C).

This suggests that the augmentation of melanogenesis by butin, caffeic acid and luteolin in zebrafish occurs via stimulation of tyrosinase activity. The proliferation of cellular tyrosinase activity by these compounds may be caused either by direct stimulation of enzyme activity or by an augmentation in the amount of tyrosinase protein in melanocytes.

### 2.4. Effects of Anti-Inflammatory and Inhibition of Reactive Oxygen Species

As explained previously, ethanol seed extract of *Vernonia anthelmintica* exhibits significant in vitro antioxidant and in vivo anti-inflammatory potential. Therefore, we investigated whether these three compounds of butin, caffeic acid and luteolin have such functions in vivo or not.

**Figure 7:**
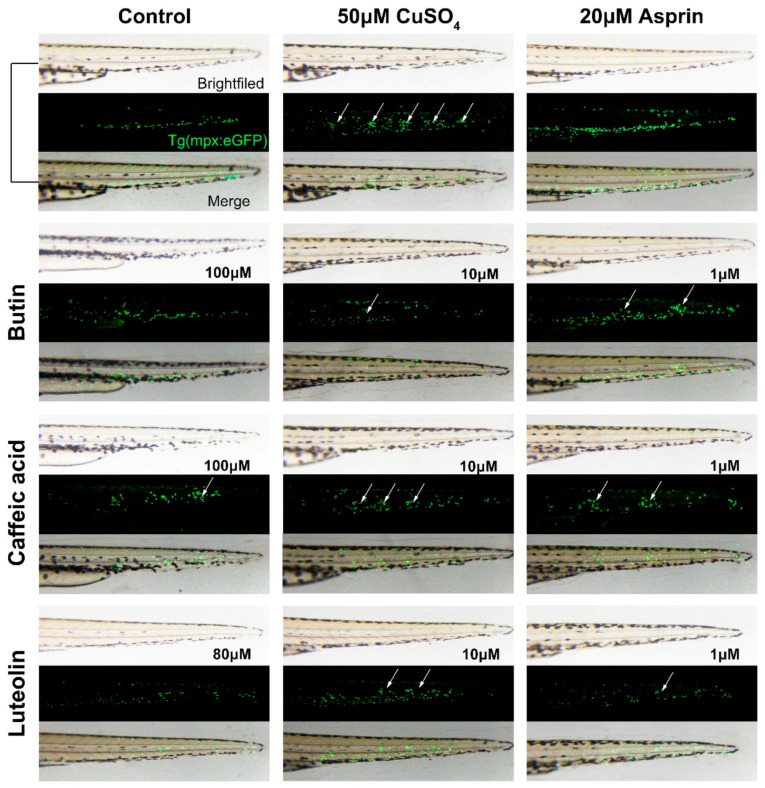
The anti-inflammatory effect of compounds in the chemically induced inflammation model. The time of image acquisition was when Tg(*mpx:GFP*) zebrafish larvae develop to 56 hpf. Currently, transgenic zebrafish larvae exhibit green fluorescent leukocytes. Untreated fish (control) show the normal distribution of labeled cells, mostly localized in the ventral trunk and tail. In copper-treated siblings (50 μM CuSO_4_), leukocytes become localized preferentially to a few clusters along the horizontal midline of the trunk and tail (see white arrows); no overt tissue damage to the larvae is observed in bright-field images., and relieving inflammation by applying aspirin (20 μM) as a positive standard. Before treated with CuSO_4_, zebrafish larvae were incubated with butin and caffeic acid [1, 10, 100] μM.

As the results show in Figure 7, 50 μM CuSO4 could induce leukocytes to become localized preferentially to a few clusters along the horizontal midline of the trunk and tail (see white arrows), meaning severe inflammation in Tg(mpx:GFP) zebrafish larvae. When treated by butin (100 μM), caffeic acid (100 μM) and luteolin (80 μM), the inflammation was effectively suppressed, and the number of leukocytes bundle significantly reduced, which results in the best luteolin, butin followed, caffeic acid worst.

**Figure 8:**
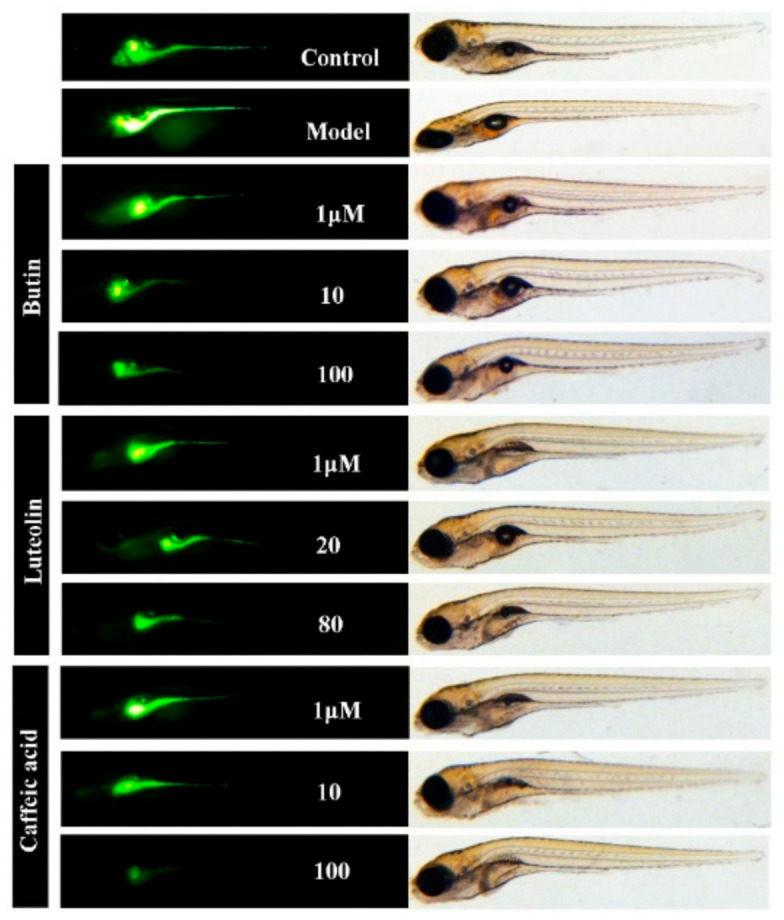
The inhibition of reactive oxygen species effect of compounds in zebrafish. Zebrafishes (5 dpf) were treated with different doses of butin [1, 10, 100] µM, luteolin [1, 20, 80] µM and caffeic acid [1, 10, 100] µM for 24 h and then H2O2 (500 µM) for 4 h, respectively. DCFH-DA (10 µM) was added to zebrafish for 30 min, and then images were captured.

Caffeic acid has good antioxidant properties in vitro, but the antioxidant properties of butin and luteolin are unknown [24]. We used hydrogen peroxide to build a ROS-enriched zebrafish model and utilized DCFH-DA fluorescent probe to detect the antioxidant properties of the compounds. The intensity of fluorescence reflects the level of ROS in zebrafish. From our results in Figure 8, caffeic acid showed the best antioxidant, at a concentration of 100 μM, the level of ROS is even lower than normal. Butin and luteolin also have certain antioxidant properties. As expected, with increasing concentration of admin-istration, ROS levels were all reduced. Therefore, these three compounds represent certain effects of anti-inflammatory and inhibition of reactive oxygen species in vivo.

### 2.5. The Inducing Melanogenesis Activity and Combinational Design of Components

The D-optimal Design was employed to investigate the synergistic effect of three active components (butin, luteolin and caffeic acid) with varying concentrations. Each combination group is equal to concentration (100 μM). The numbers in each row indicate concentrations of three monomers. Their relative of melanin contents (% of control) mean ± s.d. was used to evaluate the effect of each component mixture group as shown in Table 1.

**Figure 9:**
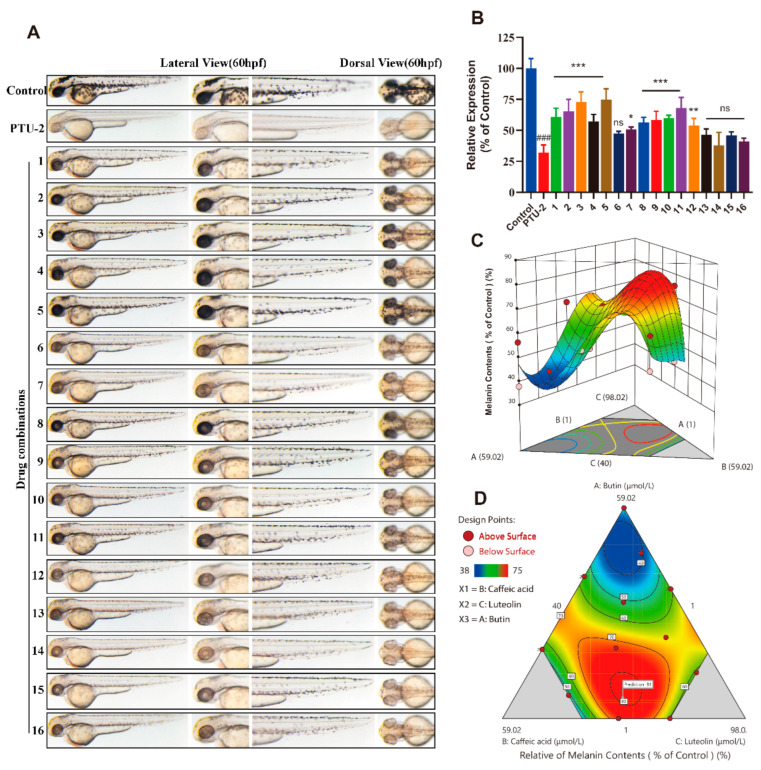
The inducing melanogenesis activity and combinational design of components, and response surface methodology and contour plots of melanogenesis effect for component combinations. (**A**) Zebrafish melanin phenotype in each combination group. (**B**) The relative of melanin contents (% of control), results shown are mean ± SEM. Data were analyzed by ANOVA followed by post hoc Tukey test. n = 30 zebrafish, ^###^ *p* < 0.001 vs. control, ns, non-significant, *** *p* < 0.001, ** *p* < 0.01, * *p* < 0.05 vs. PTU-2. (**C**) 3D response surface of the relative of melanin contents (% of control). (**D**) The results of this figure were predicted by the data reported in Table 1.

Similar to the efficacy of the monomer, we obtained the zebrafish melanin phenotype and quantitative data for each component mixture group. Compared with the PTU-2 group, the melanin levels in most of groups have a significant increase (Figure 9A,B).

Moreover, the effect of melanin content (% of control) recovery in some groups is stronger than that of the monomer, which suggests that the three compounds have a synergistic effect on inducing melanogenesis.

Application of response surface methodology (RSM) in the optimization of analytical procedures is today largely diffused and consolidated, principally because of its advantages to classical one-variable-a-time optimization, such as the generation of large amounts of information from a small number of experiments and the possibility of evaluating the interaction effect between the variables on the response [25,26]. RSM aims to find the optimal process settings to achieve peak performance. In this paper, we utilized the model Y = f (X1, X2, X3). The variables are independent variables, such that the response depends on them. The Least Square method was used to estimate the parameters in the polynomials. We used the Design Expert 12.0.3.0 to complete the above computation process. The results are shown in Figure 9C,D. From the mathematic model, we can get a predicted best melanin content (% of control) recovery rate of 81% by combination of butin, caffeic acid and luteolin (butin:caffeic acid:luteolin = 7.38:28.30:64.32) rather than mono-compounds.

### 2.6. Validation of Mathematical Model of Drug Combination

**Figure 10:**
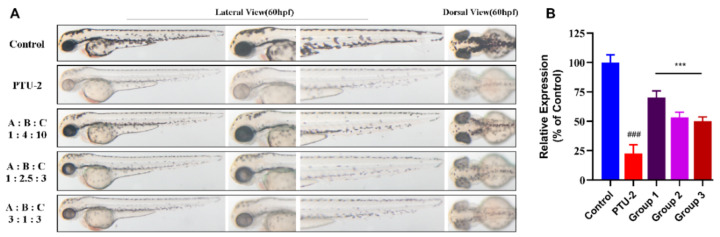
Validation of mathematical model of drug combination. (**A**) Zebrafish melanin phenotype in various ratio of compounds (butin-A, caffeic acid-B, and luteolin-C). Each combination group is equal in concentration (100 μM). (**B**) Collecting zebrafish in each group for testing relative expression (% of control). Group 1, 2 and 3 mean ratio (1:4:10, 1:2.5:3 and 3:1:3) groups. Results shown are mean ± SEM. Data were analyzed by ANOVA followed by post hoc Tukey test. n = 30 zebrafish, ^###^ *p* < 0.001 vs. Control, *** *p* < 0.001 vs. PTU-2.

In order to verify the reliability of the model, we selected the predicted optimal solution (butin:caffeic acid:luteolin = 1:4:10) and the non-optimal solution (butin:caffeic acid:luteolin = 1:2.5:3 and 3:1:3) to verify the effect of melanin synthesis. As the results shown in Figure 10A,B, compared with the latter, the predicted optimal solution has the highest relative melanin content (% of control), although the predicted value has a certain deviation, which confirmed that the model has certain credibility.

### 2.7. Combination Compounds Enhance the Expression Level of Melanogenic Genes

Melanogenesis is regulated by melanogenic enzymes including tyrosinase (TYR), tyrosinase-related protein 1 (TRP 1) and tyrosinase-related protein 2 (TRP 2) [27,28]. TYR directly mediates the production of melanin via the oxidation of melanogenic substrates tyrosine [29]. Microphthalmia-associated transcription factor (MITF) is a master regulator of the transcription of genes involved in melanin synthesis. In the process of differentiation of neural crest cells into melanocytes, gene *mitfa*, as one of its earliest occurrence markers, plays a core regulatory role in the development and differentiation of melanocytes, and it also plays an important role in the formation of zebrafish pigment [30,31,32]. During the early development of zebrafish body color, gene *dct* and *kit*, they promote the differentiation and migration of melanocyte stem cells into melanocytes [33,34].

**Figure 11:**
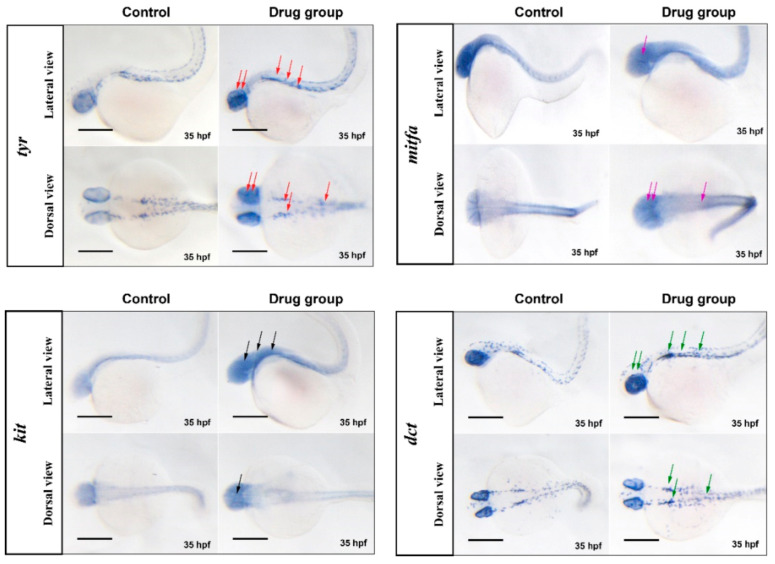
Combination compounds enhance the expression level of melanogenic genes. By the method of in situ hybridization on whole-mount zebrafish embryos to investigate the effects of the best drugs combination on the expression of melanogenic genes (*tyr*, *mitfa*, *kit* and *dct*). Zebrafish larvae were incubated with combination of butin, caffeic acid and luteolin (butin:caffeic acid:luteolin = 7.38:28.30:64.32). In 35 hpf, collecting zebrafish and hybridizing with labeled nucleic acid probes. The color rendering (blue) is means expression of specific genes, the red, purple, black and green arrows indicate the expression sites of tyr, mitfa, kit, and dct, respectively. The scale bars represent 300 μm.

Therefore, we utilized the method of in situ hybridization on whole-mount zebrafish embryos to investigate the effects of the best drugs combination on the expression of melanogenic genes (*tyr*, *mitfa*, *kit* and *dct*) during the development of zebrafish (35 hpf). In 35 hpf, all the genes are expressed in the eyes and dorsum of zebrafish larvae, small amount of tail and almost no abdomen (Figure 11). Contrasted to control group, the combination improves the expression level of *tyr*, *kit* and *dct* in the eyes and dorsum of zebrafish larvae (see red, black and green arrows). However, the expression level of *mitfa* is only slightly increased in the dorsum (purple arrows), and it may be related to the time course of melanocyte development in the eyes and dorsum.

## 3. Discussion

In this study, we attempted to prove that the extract of *Vernonia anthelmintica (L.) Willd.* can enhance the melanin synthesis function of B16F10 cells in vitro and confirmed that it can be used to treat vitiligo. We found that butin, caffeic acid and luteolin have a certain melanogenesis effect, of which butin is the best. However, the results in vitro are not particularly stable, which is related to the state of the cells, which leads to changes in melanin content. In addition, melanogenesis is closely related to the microenvironment of melanocytes and in vitro studies cannot reflect this feature. Hence, we chose the holistic-model-organism zebrafish to confirm the excellent melanogenesis effects of three compounds. In addition, previous employment suggested that the extract of *Vernonia anthelmintica (L.) Willd.* equally efficient [35]. Caffeic acid and luteolin are the first we discovered that have biological functions in melanin synthesis, although the effect is not extraordinarily strong.

As is well known, vitiligo is a multifactorial disease, with both genetic and environmental factors implicated in its initiation [36]. The same causal mechanisms might not apply to all cases, and different pathogenetic mechanisms might work together, ultimately leading to the same clinical result. Progression and maintenance of vitiligo were identified to be related with numerous inflammatory signaling pathways [37]. Previously, melanocytes are particularly susceptible to stress because they perform melanogenesis, accompanying mitochondrial energy metabolism generating ROS [38]. Finally, the process of melanogenesis itself liberates hydrogen peroxide, a ROS precursor. This kind of cellular stress in melanocytes activate the innate immune system through the generation and release of DAMPs, which provide the initiating danger signal [39]. The inflammation that ensues ultimately leads to activation of the adaptive immune system, thereby facilitating autoimmune destruction and vitiligo progression [36]. Therefore, relieving melanocytes in oxidative stress may become a treatment strategy for vitiligo. Consequently, our work confirmed that butin, caffeic acid and luteolin have superior properties on anti-inflammatory and antioxidant.

As a traditional Chinese medicine, *Vernonia anthelmintica (L.) Willd.* extract is proven to be an effective drug in curing cutis disease, but its component range and working mechanism remain unknown [40]. In addition, traditional Chinese medicine (TCM) usually takes effect when multiple components play a synergistic role at the same time. It previously confirmed that butin, caffeic acid and luteolin in promoting melanogenesis, anti-inflammatory and antioxidant capacity have their own advantages. Furthermore, *Vernonia anthelmintica (L.) Willd.* extract is more effective than any monomer in promoting melanogenesis, which means that there is a synergistic effect between the compounds. To optimize the proportion of the three mono-compounds to exert the maximum pharmacological effect, we adopted the mathematical model called the D-Optimal Design and RSM method (Figure 9). The best melanin content recovery rate (of 81%) is achieved with a butin:caffeic acid:luteolin ratio of 7.38:28.30:64.32. Subsequently, we validated the accuracy of our current result, but we will continue to use the mathematical model to balance the varied positive and negative effects with the overall disease curing purpose.

Finally, we utilized the method of in situ hybridization on whole-mount zebrafish embryos to investigate the effects of the best drugs combination on the expression of melanogenic genes (*tyr*, *mitfa*, *kit* and *dct*). In Figure 11, the combination enhances *tyr*, *kit* and *dct* expression on eyes and dorsum of zebrafish larvae, and *mitfa* on dorsum. It indirectly proved that the compounds could promote the proliferation, differentiation and melanin synthesis of melanocytes.

Consequently, our work confirmed that *Vernonia anthelmintica (L.) Willd.* extract has melanogenesis-promoting capacity in vivo. We also confirmed and indicated that the mono-compounds may be responsible for the seed extract the capacity. We also showed that butin, caffeic acid and luteolin have promoting melanogenesis, anti-inflammatory and antioxidant effects. We used a combination of pharmacological and statistical techniques to form a suitable model for developing drug combinations. Furthermore, we have indicated the best combination of mono-compounds present in *Vernonia anthelmintica (L.) Willd.* extract that is responsible for melanogenesis activity. Hence, this study forms the basis for future research on these pharmacologically active compounds and confirms their effectiveness. This study also uses methodologies that can be helpful in the future modernization of TCMs during modern drug development procedures.

## 4. Materials and Methods

### 4.1. Reagents

Butin was prepared by the Pharmaceutical Chemistry Laboratory of China Pharma-ceutical University. Caffeic acid and luteolin are purchased from Aladdin industrial corporation (Shanghai, China). α-Melanocyte-stimulating hormone (α-MSH), phenyl-thiourea (PTU) and hydrogen peroxide were purchased from Sigma-Aldrich (USA). Dimethyl sulfoxide (DMSO), _L_-3,4-dihydroxyphenylalanine (_L_-DOPA) were purchased from Sigma-Aldrich (MO, USA). Dulbecco’s modified Eagle’s medium (DMEM) was purchased from GIBCO(USA), FBS (Hyclone, Australia). Fontana-Masson Stain Kit was purchased from SenBeiJia Biological Technology Co., Ltd. (Nanjing, China).

### 4.2. Cell Culture

The melanoma cell line B16F10 was purchased from the Chinese Cell Bank (Shanghai, China) and maintained as a monolayer culture in DMEM supplemented with 10% (*v*/*v*) FBS, 100 U·mL^−1^ penicillin and 100 μg·mL^−1^ streptomycin (Beyotime Biotechnology, Shanghai, China) at 37 °C in a humidified 5% of CO_2_ incubator.

### 4.3. Cell Viability Assay

Cell viability was determined by MTT assay. B16F10 cells were plated in 96 well plates at a density of 3 × 10^3^ cells per well. When cells grow to 30% density, the cells were incubated with the compounds for 48 h, the culture medium was removed and replaced with 20 mL of MTT solution (5 mg·mL^−1^) dissolved in fresh DMEM and incubated for 4 h. The medium was removed completely, and 150 µL of DMSO was added to each well and fully dissolved for 5 min. Optical absorbance was set at 570 nm with a microplate spectrophotometer (, New Jersey, USA). Absorbance of cells without treatment was considered as 100% of cell survival. Each treatment was performed in six multiple holes.

### 4.4. Masson-Fontana Melanin Ammonia Silver Stain

B16F10 cells with a growth density of about 40% in a 6-well plate were added with different concentrations of test drugs and cultured at 37 °C and 5% CO_2_ for 48 h. Fontana-Masson Stain Kit was used for melanin staining. The melanin content was observed by NIKON Upright Fluorescence Microscope.

### 4.5. Maintenance of Zebrafish

Zebrafish (*Danio rerio*) farming was carried out in accordance with methods detailed in “The Zebrafish Book” with a 14 h light (8:00 a.m. to 10:00 p.m.) and 10 h dark (10:00 p.m. to 8:00 a.m.) cycle at a temperature of 28 °C; animals were fed twice a day (9:00 a.m. and 5:00 p.m.) [41]. The experiments were started at 8:00 p.m. by placing one female and one male in the spawning tank, divided by a separator plate. The following day, the lights were turned on at 8: 00 a.m. and the separator plate was removed for 20 min. The zebrafish were then returned to their respective tanks, and the embryos in the spawning tank were collected and cleaned before they were cultured in zebrafish embryo medium and placed in an illuminator at 28 °C.

### 4.6. Melanogenesis and TYR Activity in zebrafish(or Cell)

To characterize the melanogenic effects of these compounds, total melanin content of whole zebrafish extracts was measured. The effect of these compounds on the pigmentation of zebrafish embryos was determined according to a previous report [42]. The embryos were pre-treated with 0.2 mmol·L^−1^ PTU from 6 hpf to 30 hpf (24 h). PTU was then removed, embryos were immediately washed and treated with different concentrations of the test drugs or not (PTU-2); untreated embryos were used as a control and kept undisturbed for the next 24 h, up to 54 hpf. Phenotype-based evaluation of body pigmentation was performed at 54 hpf. Embryos were anesthetized and photographed under SZX16 stereo microscope. The levels of intracellular and secreted melanin were measured as described previously.

The levels of intracellular and secreted melanin were measured by NaOH hydrolysis method. Total melanin in the cell pellet was dissolved in 100 μL of 1N NaOH/10% DMSO for 1 h at 80 °C. The absorbance at 405 nm was measured. Melanin content was calculated as a percent of the control. Specific melanin content was adjusted by the amount of protein in the same reaction.

TYR activity and melanin content assay was carried out according to a previous report with slight modifications [35]. TYR activity in zebrafish was examined by measuring the rate of oxidation of _L_-DOPA. Briefly, the zebrafish were treated with a test compound; after 54 hpf they were washed with ice-cold PBS and lysis was performed by incubation in cell lysis buffer at 4 °C for 20 min. After sonication, the lysates were then centrifuged at 14, 000 rpm for 15 min. Tyrosinase activity was then determined as follows: 100 mL of supernatant containing total 20 μL of centrifuged proteins was added to each well in a 96-well plate, and then mixed with 100 mL 0.1% _L_–DOPA in 0.1 M PBS (pH 6.8) (M/V). After incubation at 37 °C for 0.5 h, dopachrome formation was monitored by measuring absorbance at 475 nm. Specific tyrosinase activity was normalized with protein content in the reaction.

### 4.7. Chemically Induced Inflammation Assay in zebrafish

On the night before delivery, healthy and mature spotted macrophage fluorescent zebrafish were placed in the breeding tank. The embryos were collected on the second day and incubated in a constant temperature light incubator.

At 56 hpf, the hatched larvae are divided into six-well plates with 10–20 tails per well. A control group, a model group and a drug group are set up. The control group is not treated, and the drug group is first infiltrated with liquid. After 1 h, the drug solution was washed, the model group and the administration group were simultaneously added with 50 mM copper sulfate solution to induce inflammation. After 40 min, they were taken out and immediately photographed to observe the aggregation of zebrafish nerve mound macrophages [43].

### 4.8. Antioxidative Activities in Zebrafish with DCFH-DA Fluorescent Probe

Zebrafish embryos were collected and add 0.2 mmol/L PTU solution added at 6 hpf. Soaked until the zebrafish develop 120 hpf, then washed with the PTU solution, a certain number of zebrafish were soaked in the medicated egg water for 48 h, washed with liquid, and made a model with hydrogen peroxide (1 mmol·L^−1^) for 4 h. After washing out the hydrogen peroxide solution, zebrafish were soaked in the diluted DCFH-DA fluorescent probe solution (10 μmol·L^−1^), in the dark for 0.5 h, and then take pictures with a stereo microscope.

### 4.9. In Situ Hybridization on Whole-Mount Zebrafish Embryo

The whole embryo in situ hybridization technique used here mainly refers to the 2008 version of Thisse Laboratory. Before the whole zebrafish embryo hybridization in situ, the design of antisense RNA probe is the key. Using gene cloning and PCR technology, total RNA extraction, reverse transcription into cDNA, primer design and PCR amplification with primers. Then it was connected to the vector, transformed, cloned, identified and sequenced. Finally, four genes, *tyr*, *mitfa*, *kit* and *dct*, were successfully cloned and identified, and antisense RNA probes were prepared. The primers used were:

*tyr* (forward 5′-AGGGTTCTGTCAGGACGTC-3′, reverse 5′-GACTCTACATCGGCGGATG-3′)
*kit* (forward 5′-ACTGTTCGGCTTGTTCCA-3′, reverse 5′-CGATGAGCAGCGGT-GTAA-3′)
*mitfa* (forward 5′-AGGGTTCTGTCAGGACGTC-3′, reverse 5′-GA-CTCTACATCG-GCGGATG-3′)
*dct* (forward 5′-AGGGTTCTGTCAGGACGTC-3′, reverse 5′-GACTCTACATCGGCGGATG-3′).

Briefly, zebrafish embryos were collected, synchronized and arrayed by pipette into a 6-well plate. Each well was filled with 40 embryos along with 5000 µL of zebrafish embryo medium. When the embryo develops to 9 hpf, one of the holes is selected to give the combination drug, and one hole is used as the blank control group, and the embryos at the 35 hpf period are collected. Each group of embryos is divided into 4 EP tubes, and 10 embryos in each tube. Replace the solution with fresh fixative 4% PFA. Repeat 3 or 4 times. Finally, end up with 100% fixative. Generally, the fixed embryos will be placed at 4 °C overnight to allow the fixation process completely. Dehydrate embryos with methanol and rehydration with PBST step by step. Remove the washing PBST, add new fresh PBST to 1.5 mL, add the desired proteinase K directly into the tube and gently roll the tube to increase the proteinase K diffusion. (Vortex the tube and make sure the precipitate be mixed evenly before use). After that, post-fix, hybridize wash, block, immnohybridize and visualize. The embryos were observed by NIKON Upright Fluorescence Microscope.

### 4.10. Drug Combination

To further discover the synergistic effect of the mono-compounds, a D-optimal Design was employed to arrange the experiment. The details regarding D-optimal Design are described in many references. A Response Surface Methodology (RSM) method was utilized to optimize the combination of butin, caffeic acid and luteolin. We used the software Design-Expert^®^ 12 to analyze the whole process.

### 4.11. Statistical Analysis

All data were expressed as mean ± standard error. Statistical analysis was performed with a one-way ANOVA followed by Tukey’s post hoc test for correction of multiple comparisons. Values with *p* < 0.05 were considered significant.

## Figures and Tables

**Table 1:** The Contents are the relative of melanin contents (% of control group), and the combinations are concentrations (μmol/L) of the three compounds. Combinations in D-optimal Design and their relative of melanin contents (% of control) mean ± s.d.

Group	Butin	Caffeic Acid	Luteolin	Contents (%)
1	20.02	40.00	40.00	60.67 ± 5.86
2	23.34	8.82	67.86	65.39 ± 7.83
3	20.43	22.22	57.37	73.02 ± 6.47
4	40.14	19.88	40.00	57.11 ± 4.65
5	1.00	31.30	67.72	74.92 ± 6.97
6	36.66	1.00	62.36	47.5 ± 1.39
7	33.02	14.09	52.91	50.72 ± 1.58
8	59.02	1.00	40.00	56.39 ± 3.34
9	1.00	19.02	80.00	58.23 ± 5.8
10	13.68	6.34	80.00	59.78 ± 1.92
11	36.66	1.00	62.36	68.06 ± 6.91
12	40.14	19.88	40.00	53.8 ± 4.75
13	7.38	40.00	52.64	46.43 ± 3.83
14	59.02	1.00	40.00	37.81 ± 8.67
15	20.02	40.00	40.00	46.02 ± 2.26
16	46.64	3.08	50.31	41.09 ± 2.03

## Data Availability

The data presented in this study are available on request from the corresponding author.
